# Targeting cytosolic phospholipase A_2_ α in colorectal cancer cells inhibits constitutively activated protein kinase B (AKT) and cell proliferation

**DOI:** 10.18632/oncotarget.2639

**Published:** 2014-10-28

**Authors:** Zhong Zheng, Xiangyi He, Chanlu Xie, Sheng Hua, Jianfang Li, Tingfeng Wang, Mu Yao, Soma Vignarajan, Ying Teng, Leila Hejazi, Bingya Liu, Qihan Dong

**Affiliations:** ^1^ Department of Gynecologic Oncology, Fudan University Shanghai Cancer Center, Department of Oncology, Shanghai Medical College, Fudan University, Shanghai, China; ^2^ Department of Gastroenterology, Ruijin Hospital, Shanghai Jiaotong University School of Medicine, Shanghai, China; ^3^ Central Clinical School and Bosch Institute, The University of Sydney and Department of Endocrinology and Sydney Cancer Centre, Royal Prince Alfred Hospital, Sydney, Australia; ^4^ Shanghai Key Laboratory of Gastric Neoplasms, Department of Surgery, Shanghai Institute of Digestive Surgery, and Gastroenterology, Ruijin Hospital, Jiaotong University School of Medicine, Shanghai, China; ^5^ Department of General Surgery, Nanhui Central Hospital. Shanghai, China; ^6^ School of Science and Health, The University of Western Sydney, Australia

**Keywords:** Cytosolic phospholipase A_2_α, AKT, Colorectal cancer, Efipladib

## Abstract

A constitutive activation of protein kinase B (AKT) in a hyper-phosphorylated status at Ser^473^ is one of the hallmarks of anti-EGFR therapy-resistant colorectal cancer (CRC). The aim of this study was to examine the role of cytosolic phospholipase A_2_α (cPLA_2_α) on AKT phosphorylation at Ser^473^ and cell proliferation in CRC cells with mutation in *phosphoinositide 3-kinase (PI3K).* AKT phosphorylation at Ser^473^ was resistant to EGF stimulation in CRC cell lines of DLD-1 (*PIK3CA^E545K^* mutation) and HT-29 (*PIK3CA^P499T^* mutation). Over-expression of *cPLA_2_α* by stable transfection increased basal and EGF-stimulated AKT phosphorylation and proliferation in DLD-1 cells. In contrast, silencing of *cPLA_2_α* with siRNA or inhibition with Efipladib decreased basal and EGF-stimulated AKT phosphorylation and proliferation in HT-29. Treating animals transplanted with DLD-1 with Efipladib (10 mg/kg, *i.p.* daily) over 14 days reduced xenograft growth by >90% with a concomitant decrease in AKT phosphorylation. In human CRC tissue, cPLA_2_α expression and phosphorylation were increased in 63% (77/120) compared with adjacent normal mucosa determined by immunohistochemistry. We conclude that cPLA_2_α is required for sustaining AKT phosphorylation at Ser^473^ and cell proliferation in CRC cells with *PI3K* mutation, and may serve as a potential therapeutic target for treatment of CRC resistant to anti-EGFR therapy.

## INTRODUCTION

Colorectal cancer (CRC) is the third most commonly diagnosed cancer in males and the second in females worldwide with over 1.2 million new cases annually [1]. Due to the lack of effective treatment for metastatic CRC, there are approximately 600,000 deaths annually [1]. Despite the improvement in the clinical outcome following the development of molecular targeted therapy against the epidermal growth factor receptor (EGFR) [2], CRC with mutations of *BRAF*, *RAS, PI3K or PTEN* are resistant to anti-EGFR therapy [3, 4]. *RAS* and *PIK3CA* mutation increased protein kinase B (AKT) phosphorylation at Ser^473^ [5]. Phosphorylation of AKT at Ser^473^ is required for tumor progression in colon cancer [6]. Therefore, a constitutive activation of AKT in a hyper-phosphorylated status at Ser^473^ is one of the hallmarks of anti-EGFR therapy-resistant CRC [7]. Hence, identification of pathways that are required for maintaining AKT phosphorylation at Ser^473^ in CRC is of clinical importance.

Previous studies have shown the involvement of prostaglandin and its producing enzyme cyclooxygenase (COX) in CRC [8, 9]. The enthusiasm for the effectiveness of COX-2 inhibitor is hampered by its side effect due to the selective inhibition of COX enzymes. Phospholipase A_2_ (PLA_2_) is a family of enzymes that catalyse the hydrolysis of fatty acid at the *sn*-2 position of glycerophospholipid on cell membranes [10]. Of the family members, cytosolic PLA_2_α (cPLA_2_α) is the only enzyme that catalyses the specific hydrolysis of arachidonic acid (AA) [10]. The cleaved free AA is converted to eicosanoids by the COX and lypoxygense (LOX) enzymes [10]. As the inhibition of cPLA_2_α reduces the supply of AA to both COX-1 and COX-2 enzymes, it may avoid the side effect of selective COX-2 inhibitors. Moreover, 5-LOX is over-expressed in CRC compared with normal colonic mucosa [11]. Blocking 5-LOX reduces CRC cell proliferation *in vitro* and *in vivo* [11]. Hence, we have evaluated the potential using cPLA_2_α as a therapeutic target for treatment of CRC. This paper describes the effect of ectopic expression, genetic silencing or pharmacological inhibition of cPLA_2_α on AKT phosphorylation at Ser^473^ and cell proliferation *in vitro* and *in vivo* of CRC cells with constitutive activation of AKT due to gain-of-function mutations in *PI3K*, as well as cPLA_2_α expression and activation in human CRC tissues.

## RESULTS

### Over expression of cPLA_2_α elevates basal and EGF-stimulated phospho-AKT levels at Ser^473^ with parallel increase in proliferation of CRC cells with *PIK3CA^E545K^* mutation

**Figure 1:**
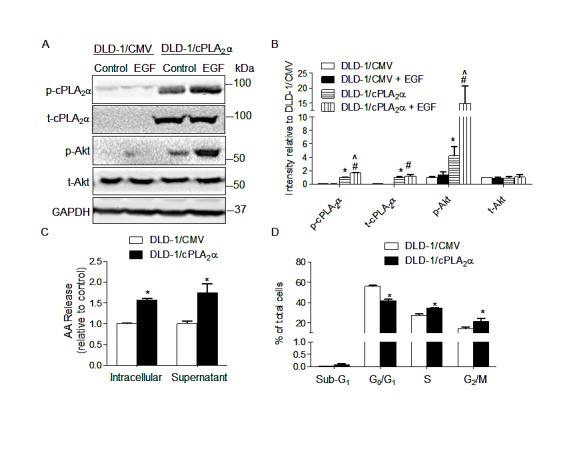
Overexpression of cPLAα increases p-AKT and cell proliferation in DLD-1 cells (A) Immunoblot in DLD-1 cells stably transfected with cPLA_2_α (DLD-1/cPLA_2_α) or empty vector (DLD-1/CMV) with or without EGF treatment (20 ng/mL, 30 min). (B) Densitometry quantification of (A). ^*^P<0.05 vs. DLD-1/CMV, ^#^*P*<0.05 vs. DLD-1/CMV+EGF, ^^^*P*<0.05 DLD-1/cPLA_2_α vs. DLD-1/cPLA_2_α+EGF, n=3. (C) Arachidonic acid concentration in intracellular compartments and the supernatant measured by Mass Spectrometer. ^*^P <0.05 vs. DLD-1/CMV. (D) DNA content analysis by PI-Flow cytometry. ^*^P <0.05 vs. DLD-1/CMV, n=3. All data was expressed as Mean ± SD.

To determine the effect of over expression and activation of cPLA_2_α on AKT phosphorylation at Ser^473^ and cell proliferation, DLD-1 cells (*PIK3CA^E545K^*) were stably transfected with cPLA_2_α-coding vector (DLD-1/cPLA_2_α) or empty vector (DLD-1/CMV). The ectopically expressed cPLA_2_α led to an increase in total (t-cPLA_2_α) and phospho-cPLA_2_α at Ser^505^ (p-cPLA_2_α, Figure 1A and 1B), with a concomitant increase in arachidonic acid levels in the intracellular and extracellular (medium) compartments (Figure 1C).

Basal and EGF (final concentration 20 ng/mL, 30 min) stimulated p-AKT at Ser^473^ was increased 3.2-fold (Figure 1A: DLD-1/cPLA_2_α without EGF *vs.* DLD-1/CMV without EGF) and 9.5-fold (DLD-1/cPLA_2_α with EGF *vs.* DLD-1/CMV with EGF), respectively in DLD-1/cPLA_2_α compared with DLD-1/CMV cells (both *P*<0.001, Figure 1B). Levels of p-AKT at Ser^473^ were unchanged in the presence or absence of EGF stimulation in DLD-1/CMV cells (Figure 1A: DLD-1/CMV without EGF *vs.* DLD-1/CMV with EGF). However, the same dose of EGF elicited a distinct increase in p-AKT in DLD-1/cPLA_2_α cells (DLD-1/cPLA_2_α without EGF *vs.* DLD-1/cPLA_2_α with EGF, *P*<0.05). Levels of t-AKT were unaffected in both cell lines in the presence or absence of EGF stimulation. It is interesting to note that, similar to p-AKT at Ser^473^, p-cPLA_2_α at Ser^505^ levels remained unchanged in DLD-1/CMV cells in response to EGF stimulation (Figure 1A). However, EGF elicited a marked increase in p-cPLA_2_α at Ser^505^ in DLD-1/cPLA_2_α cells (*P*<0.05, Figure 1A and 1B), while the levels of t-cPLA_2_α remained unchanged. Cell cycle phase distribution analysis showed that the proportion of G_1_/G_0_ was lower, whereas S and G_2_/M were higher, in DLD-1/cPLA_2_α than DLD-1/CMV cells (all *P*<0.05, Figure 1D), with no significant change in the proportion of cells in sub-G_1_ phase.

### Silencing of cPLA_2_α decreases EGF-stimulated phospho-AKT at Ser^473^ levels and proliferation in CRC cells with mutant *PIK3CA^P499T^*

**Figure 2:**
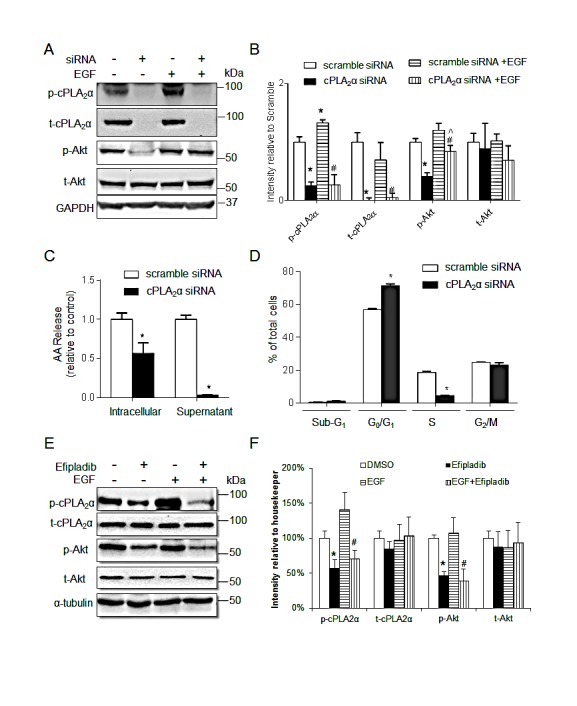
Silence of cPLAα decreases EGF-stimulated p-AKT and cell proliferation in HT-29 cells (A) immunoblot and (B) quantification of cPLA_2_α and AKT in cells transfected with cPLA_2_α siRNA or scramble control (10 nM for 72 h) with or without EGF treatment (20 ng/mL, 30 min). ^*^P <0.05 vs. cells transfected with scramble control without EGF; ^#^*P* <0.05 vs. cells transfected with scramble control with EGF. ^^^*P* <0.05 cPLA_2_α siRNA vs. cPLA_2_α siRNA+EGF. (C) Arachidonic acid concentration in the intracellular and supernatant compartments measured by Mass Spectrometry. ^*^P <0.05 vs. cells transfected with scramble control. (D) DNA content analysis by PI-Flow cytometry. ^*^P <0.05 vs. cells transfected with scramble control.; (E) Immunoblot of HT-29 cells treated with 25 μM Efipladib for 72 h and/or 20 ng/mL EGF for 30 min before harvesting. (F) Densitometry quantification. ^*^P <0.05 vs. DMSO, ^#^*P* <0.05 vs. DMSO+EGF, n=3. All data expressed as Mean ± SD.

We next determined the effect of genetic silencing of cPLA_2_α with siRNA on p-AKT levels and cell proliferation. Transfection of HT-29 (*PIK3CA^P499T^*) with cPLA_2_α siRNA abolished the t-cPLA_2_α and p-cPLA_2_α protein levels (all *P*<0.001, Figure 2A and 2B), and significantly decreased both intracellular and extracellular content of arachidonic acid (both *P*<0.001, Figure 2C).

Levels of p-AKT remained unchanged in response to EGF stimulation (final concentration 20 ng/mL, 30 min) in HT-29 (Figure 2A:scramble siRNA with EGF *vs.* scramble siRNA without EGF). However, Knockdown of cPLA_2_α deceased both basal and EGF stimulated p-AKT levels by 59% (Figure 2A: cPLA_2_αsiRNA without EGF *vs.* scramble siRNA without EGF) and 30% (cPLA_2_α siRNA with EGF *vs.* scramble siRNA with EGF), respectively, compared with the scrambled control (all *P*<0.05, Figure 2B). The levels of t-AKT were unchanged with or without EGF stimulation in the presence or absence of cPLA_2_α siRNA. It indicates that the constitutively-activated AKT as the results of *PIK3CA^P499T^* mutation could be inhibited by knockdown cPLA_2_α expression. Again, EGF treatment elicited an increase in p-cPLA_2_α (*P*<0.05) without affecting t-cPLA_2_α when endogenous cPLA_2_α was unperturbed (Figure 2A and 2B).

Next, we assessed the effect of transient knockdown of cPLA_2_α on cell cycle distribution. There was a clear increase in G_1_/G_0_ and corresponding decrease in S phase (all *P*<0.05, Figure 2D), with no significant change in the proportion of cells in sub-G_1_ phase following genetic silencing of cPLA_2_α. We then examined whether Efipladib (a new indole derived cPLA_2_α inhibitor [12, 13]) mimics the impact of cPLA_2_α siRNA and exerts the same action on AKT phosphorylation in HT-29 cells. Incubation of HT-29 cells with Efipladib (25 μM, 72 h) indeed decreased basal and EGF-stimulated p-AKT levels without affecting t-AKT (both *P*<0.05, Figure 2E and 2F). Taken together, targeting cPLA_2_α by genetic silencing or pharmacological inhibition supresses EGF-resistant AKT phosphorylation at Ser^473^ and also inhibits cell proliferation in HT-29 cells harbouring mutation in *PIK3CA^P499T^*.

### Pharmacological inhibition of cPLA_2_α decreases cell proliferation in both DLD-1 and HT-29 cells

**Figure 3:**
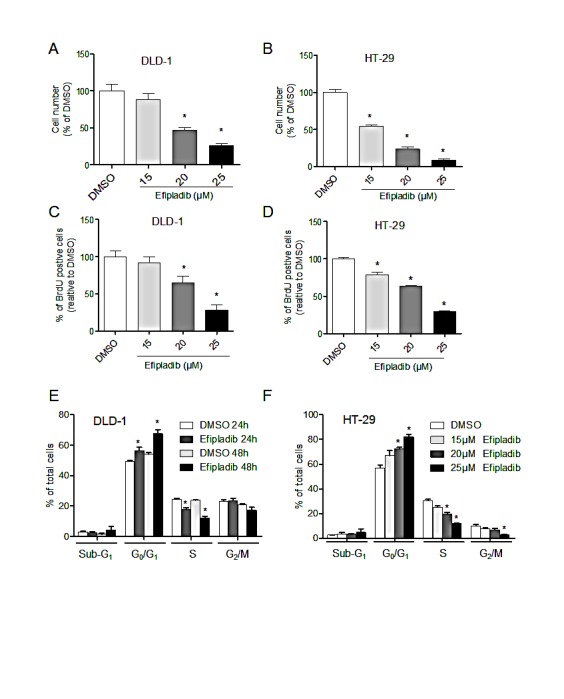
Pharmacological blockade of cPLAα by Efipladib results in decreased cell proliferation DLD-1 (A) or HT-29 cells (B) were plated in 96-well plates and treated with vehicle control (DMSO) or Efipladib for 72 h. The viable cell number was determined by the MTS assay. DLD-1 (C) or HT-29 (D) cells were plated in 6-well plates and treated with control (DMSO) or Efipladib for 72 h. BrdU was added for 3 h prior to harvesting. BrdU incorporation was determined by immunocytochemistry. Percentage of BrdU positive cells was determined as the average of 10 high-power fields (X40) per sample. ^*^P <0.05 *vs.* vehicle-treated control, n=3. (E) DLD-1 cells were treated with Efipladib at 25 μM for 1 or 2 days, followed by staining with PI and subsequent analysis with flow cytometry. ^*^P<0.05 *vs.* vehicle-treated control, n=3. (F) HT-29 cells were treated with Efipladib at indicated doses for 3 days, followed by PI-staining and DNA content analysis. ^*^P<0.05 vs. vehicle-treated control, n=3. All data expressed as Mean ± SD.

Since pharmacological blockade of cPLA_2_α with Efipladib effectively reduced basal and EGF-stimulated AKT phosphorylation, we determined the effect of Efipaldib on cell proliferation in unmodified parental DLD-1 (*PIK3CA^E545K^*) and HT-29 (*PIK3CA^P499T^*) cells. Inhibition of cPLA_2_α with Efipladib reduced cell number (*P*<0.05, Figure 3A and 3B) and BrdU incorporation (*P*<0.05, Figure 3C and 3D) in a dose-dependent manner in both DLD-1 and HT-29 cells. Efipladib treatment for 24-48 h blocked DLD-1 cell cycle progression as indicated by an accumulation of cells in the G_0_/G_1_ phase with a decrease in the proportion of cells in S phases (all *P*<0.05, Figure 3E). The decreased G_2_/M phase, however, did not reach statistical significance at 48 h. A similar effect on G_0_/G_1_ phase and S phases was noted in HT-29 cells treated with increasing dose of Efipladib after 72 h (all *P*<0.05, Figure 3F). The fraction of cells in G_2_/M was also decreased at the highest concentration of Efipladib (25 μM, *P*<0.05). We found no significant change in cell viability in the presence of Efipladib as assessed by sub-G_1_ (Figure 3E and 3F) and Trypan Blue exclusion (data not shown). Hence, consistent with effect of genetic silencing of cPLA_2_α, pharmacological blockade of cPLA_2_α resulted primarily in a cytostatic effect on CRC cells with *PIK3CA^E545K^* or *PIK3CA^P499T^*mutations.

### Pharmacological inhibition of cPLA_2_α reduces p-AKT levels and xenograft growth in mice transplanted with DLD-1 cells

**Figure 4:**
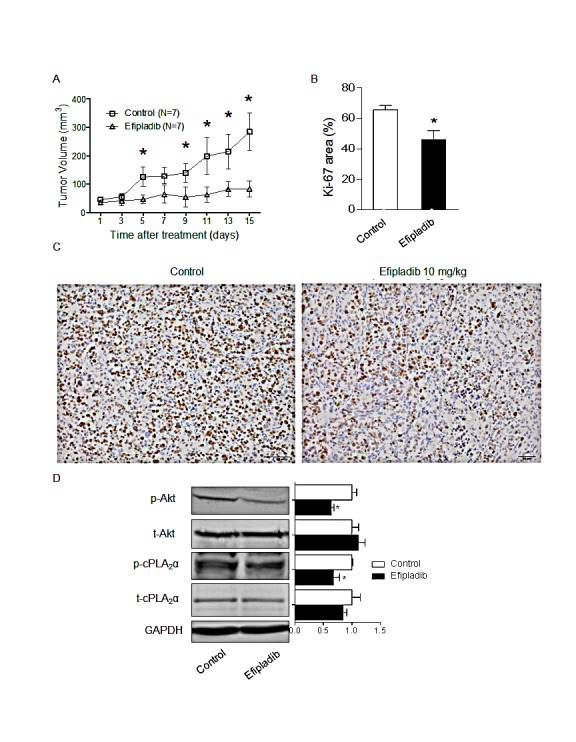
Pharmacological blockade of cPLAα by Efipladib impedes the growth of DLD-1 xenografts and decreases p-AKT levels *in vivo* (A) DLD-1 cells were inoculated into the flanks of nude mice. When xenograft tumours had reached 50 mm^3^ in volume, mice were randomised to control (n=7) or Efipladib treatment (7 mice/group) at a dose of 10 mg/kg *i.p.* daily for 14 days. Inhibition of tumour growth in the Efipladib-treated mice compared with the controls (*p*<0.001 by two way ANOVA with repeat measurement). ^*^p<0.05 vs. control at the same day. (B) The fraction of Ki-67 positive cells was determined from the average number of positive cells in 10 high-power fields (×40). *p < 0.05 vs. control. (C) Xenografts were harvested, fixed and paraffin-embedded, and stained for Ki-67 by immunohistochemistry. Scale bar = 50 μm, magnification 200×. (D) Immunoblot of DLD-1 xenograft tumour and densitometry quantification. *p<0.05 vs. control, n=3. All data expressed as Mean ± SD.

To determine if the marked decrease in p-AKT and cell proliferation in response to Efipladib can be recapitulated in animal, we treated mice carrying unmodified parental DLD-1 xenografts with Efipladib. In vehicle-treated control mice tumour volume increased 4.5-fold at day 14 compared to the day 1 (Figure 4A), but in the Efipladib–treated mice, there was only a 1.4-fold increase over 14 days (*P*<0.001 by two way ANOVA with repeat measurements). Further analysis at each time point revealed a significant difference in tumour volume as early as day 5 of Efipladib treatment (*P*<0.05, Figure 4A). Mouse body weights did not differ between the two groups. The percentage of Ki-67 positive cells and the levels of p-AKT and p-cPLA_2_α in xenografts were significantly reduced in Efipladib-treated mice compared with the vehicle-treated controls (all *P*<0.05, Figure 4B-D). The levels of t-AKT and t-cPLA_2_α remained unchanged. Hence, consistent with the *in vitro* effect of Efipladib on suppressing p-AKT and proliferation, pharmacological inhibition of cPLA_2_α *in vivo* reduces markedly p-AKT levels and DLD-1 xenograft growth compared with vehicle-treated controls.

### The levels of cPLA_2_α and phospho-cPLA_2_α at Ser^505^ are increased in colon cancer tissues

**Figure 5:**
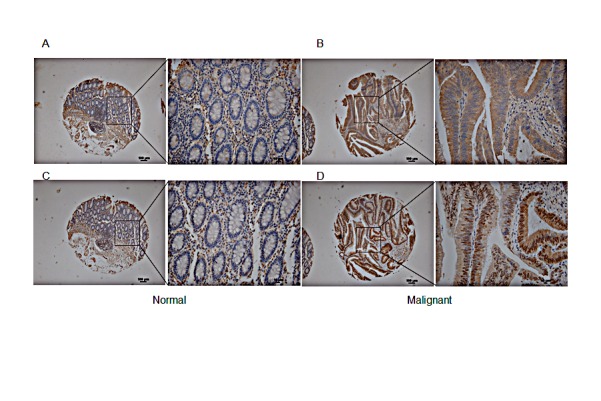
Immunohistochemical analysis of total and phospho-cPLAα at Serin human CRC tissue array Inset AC: normal colon mucosa exhibited relatively low levels of total cPLA_2_α (A) and phospho-cPLA_2_α (C). Inset BD: CRC tissue had stronger total cPLA_2_α (B) and phospho-cPLA_2_α (D) in malignant epithelial cells. Low magnification 100×. Scale bar = 100 μm. High magnification 400×. Scale bar = 10 μm.

To determine the potential of cPLA_2_α as a therapeutic target, we examined cPLA_2_α protein levels in CRC specimens by immunohistochemistry. Compared with adjacent normal epithelial cells, an increase in the extent and/or intensity of immune reactive total cPLA_2_α in malignant epithelial cells was observed in 77/120 cases (64.2%, *P*<0.001, Figure 5A and 5B). Total cPLA_2_α was mainly located in the cytoplasm in both normal and cancer cells. Although total cPLA_2_α was also present in mesenchymal cells, there was no difference between normal and cancer tissues. Among the clinical parameters analysed, total cPLA_2_α levels were correlated with poor tumour differentiation (*p*=0.029, Supplemental Table 1).

cPLA_2_α also contains several conserved serine residues as phosphorylation sites. Ser^505^ is the most studied and recognised site for phosphorylation of cPLA_2_α. Although phosphorylation is not necessary for basal enzyme activity, phosphorylation at Ser^505^ has shown to augment arachidonic acid release [14]. Immune reactive phospho-cPLA_2_α at Ser^505^ was located in nucleus and cytoplasm in both normal and cancer cells, which is consistent with previous reports in other cell types [15, 16]. An increase in the extent and/or intensity of phospho-cPLA_2_α at Ser^505^ was observed in malignant epithelial cells compared with adjacent normal epithelial cells in 76 out of 120 cases (63.3%, *P*<0.001, Figure 5C and 5D). Phospho-cPLA_2_α at Ser^505^ was also present in mesenchymal cells but not significantly different between normal and cancer. There was no association between phospho-cPLA_2_α and any tumour characteristics (Supplemental Table 1). Taken together, cPLA_2_α expression and activation are increased in nearly two thirds of CRC compared with normal mucosa.

## DISCUSSION

We provide three lines of evidence supporting the advantages of targeting cPLA_2_α in colorectal cancer.

Firstly, we have systematically investigated the role of cPLA_2_α in regulation of AKT phosphorylation by ectopic expression, genetic silencing and pharmacological inhibition in CRC cell lines with a constitutive action of AKT at Ser^473^ both in *vitro* and *in vivo*. Ectopic expression of cPLA_2_α increases basal and EGF-stimulated p-AKT levels. It is interesting to note that without manipulation of cPLA_2_α, AKT phosphorylation does not increase in response to EGF stimulation in both CRC cell lines. This is consistent with the report that constitutively-activated AKT renders cancer cells resistant to manipulation by growth factors [17]. However, a marked increase in AKT phosphorylation following EGF stimulation is noted when cPLA_2_α levels are increased by ectopic expression in DLD-1 cells. In contrast, genetic silence or pharmacological blockade of cPLA_2_α decreases basal and EGF-stimulated p-AKT levels in HT-29, which is another CRC cell line harbouring *PI3K* mutations. Same as DLD-1, p-AKT levels in HT-29 cells are resistant to EGF stimulation. Together with the effect of efipladib on p-AKT *in vivo*, these findings suggest that cPLA_2_α contributes to basal and EGF-stimulating AKT phosphorylation in CRC cells containing *PI3K* mutations. It is interesting to mention that we have shown recently that genetic silence or pharmacological blocking of cPLA_2_α decrease phospho-AKT at Ser^473^ in prostate cancer cells [18]. Hence, the link of cPLA_2_α to AKT appears to be a phenomenon not just limited to colon cancer cell lines.

The mechanism(s) by which cPLA_2_α exerts its action on AKT phosphorylation remains to be elucidated. Based on the significant change in AA concentration in response to cPLA_2_α manipulations, cPLA_2_α may exert its action on AKT *via* AA and/or its product eicosanoids [19]. Eicosanoid receptors can connect to PI3K-AKT pathway *via* heterotrimeric G proteins [20]. PGE_2_ may also be able to transactivate EGFR in CRC cells including HT-29 [21, 22]. As the decrease in proliferation of HT-29 cells by EGFR inhibitor could be abolished in the presence of PGE_2_ [23], the possible action site downstream of EGFR cannot be excluded. Furthermore, COX-2 inhibitor has been shown to increase in PTEN expression [24], which could be another mechanism for impinging on AKT. Further study is also needed to determine if cPLA_2_α can affect basal and EGF stimulated other oncogenic pathways such as ERK/MAPK.

Another interesting finding from our study is the phosphorylation of cPLA_2_α at Ser^505^, which is known to increase the AA-releasing activity [14, 25]. Previous studies have shown an increase in cPLA_2_α phosphorylation in mammalian cells by EGF [26, 27]. We found in the present study that EGF treatment increases phosphorylation of cPLA_2_α at Ser^505^ in both DLD-1 and HT-29 cells. Activation of RAS signalling by mutation or over-expression has been shown to induce PGE_2_ secretion in colon cancer [22, 28, 29]. We reported recently that AKT plays a role in stabilising cPLA_2_α protein in prostate cancer cells [30]. Hence, it appears that a self-perpetuating loop consisting of AKT and cPLA_2_α is present in CRC and maybe other type of cancer cells.

Secondly, the present study has provided evidence for the first time that pharmacological blockade of cPLA_2_α decreases cell proliferation of CRC cell lines with *PI3K* mutation both in *vitro* and *in vivo*. The presence of somatic *PI3K* mutations causing constitutive activation of AKT have been regarded as one of the predictive markers of resistance to anti-EGFR therapy [3, 4]. Therefore inhibition of constitutive activated AKT could be one of the strategies to overcome resistance to anti-EGFR therapy. Our results suggest that in addition to inhibiting AKT phosphorylation at Ser^473^, targeting *cPLA_2_α* by siRNA or inhibitor can also retard cell-cycle progression and inhibit cell proliferation in CRC cells harbouring *PI3K* mutations. Similar to Efipladib (an inhibitor of fatty acid cleavage), Cerulenin (a fatty acid synthase inhibitor) decreased AKT phosphorylation at Ser^473^, enhanced antitumor activity of oxaliplatin in human colon cancer cells [31], and suppressed liver metastasis of colon cancer in mice [32]. However, it is worth to mention that two published *in vivo* studies of cPLA_2_α in intestine or colon tumor have yielded inconsistent results. While cross-breeding of Apc^min/+^ mice with *cPLA_2_α* knockout suppresses intestine tumorigenesis [33], knockout of *cPLA_2_α* enhances azoxymethane-induced tumorigenesis in colon [34]. Hence, it is likely that azoxymethane-induced CRC may involve signalling pathways that are different from those in Apc^min/+^ mice and DLD-1 cell xenograft. The prospective of targeting cPLA_2_α is further encouraged by the report that cPLA_2_α knockout mice exhibit a relatively normal phenotype [35].

Thirdly, cPLA_2_α protein is over-expressed and hyper-phosphorylated at Ser^505^ in ~60% of colon cancer cases. The mRNA and protein levels of cPLA_2_α have been examined in CRC specimens previously. RT-PCR [36, 37], immunoblot [38] or immunohistochemistry [39-41] revealed an increased cPLA_2_α in CRC specimens, except two studies conducted by the same group reported a low cPLA_2_α expression in CRC compared to normal mucosa by immunohistochemistry [42, 43]. In the present study, we examined for the first time the levels of phospho-cPLA_2_α at Ser^505^. Although phosphorylation at Ser^505^ is not necessary for basal enzyme activity, phosphorylation at Ser^505^ has shown to augment AA release [14, 25]. In correlation with total cPLA_2_α, phospho-cPLA_2_α at Ser^505^ was clearly increased in near two-thirds of the 120 CRC specimens compared with adjacent normal mucosa. As both total and phospho-cPLA_2_α have increased in CRC, it is possible that the increase in phospho-cPLA_2_α results from the increase in total cPLA_2_α expression. Consistent with reports that activated cPLA_2_α translocates to the nucleus following stimulation with calcium ionophore or leukotriene D4 in CRC cells [44], we notice that the phospho-cPLA_2_α is present in nucleus as well, whereas total cPLA_2_α is confined in cytoplasm.

Our study suggests that poorly differentiated tumours, which is associated with unfavourable prognosis [45], are more likely having high cPLA_2_α expression. Two studies have shown that the expression of cPLA_2_α in CRC is correlated with VEGF expression but fail to predict disease-free survival and overall survival [40, 41]. *cPLA_2_α* gene polymorphisms has been shown to be associated with patients of familial adenomatous polyposis [46]. Since prognostic data of the TMA used in our study are not available, further studies are needed to determine the prognostic value of cPLA_2_α in CRC.

In summary, cPLA_2_α plays a critical role in regulation of AKT phosphorylation and cell proliferation in colon cancer cells in which *PIK3CA* has a gain-function mutation. We propose that the cPLA_2_α is a potential therapeutic target for treatment of colon cancer that are resistant to anti-EGFR therapy in the results of constitutive activation of AKT.

## MATERIALS AND METHODS

### Cell lines and Reagents

The human colon cancer cell lines DLD-1 (Cat. #: CCL-221, *PIK3CA^E545K^*,) and HT-29 (Cat. #: HTB-38, *PIK3CA^P499T^*) were purchased from the American Type Culture Collection (ATCC, Manassas, VA), and maintained in RPMI 1640 and DMEM, respectively, at 37°C in a humidified environment of 5% CO_2_. The medium was supplemented with 10% (v/v) fetal calf serum (FCS, ICN Biomedical, Irvine, CA) and all experimental cells were mycoplasma-negative. The expression plasmid pCMV6 carrying a full-length cPLA_2_α cDNA was purchased from Origene Technologies (Rockville, MD). Analytically pure Efipladib was synthesized at Sanmar Chemical, India. Antibodies against cPLA_2_α (Cat. #: SC-454) and phospho-cPLA_2_α at Ser^505^ (Cat. #: SC-34391), AKT (Cat. #: SC-8312) and phospho-AKT at Ser^473^ (Cat. #: SC-7985) were purchased from Santa Cruz Biotechnology, Inc. (Dallas, TX); Anti-Ki-67 (Cat. #: RM-9106) was from Thermo Fisher Scientific (Scoresby, VIC, Australia); Human EGF (Cat. #: E9644), BrdU (Cat. #: B5002), propidium iodide (Cat. #: P4170), antibody against BrdU (Cat. #: B8434) were from Sigma-Aldrich (St. Louis, MO). MTS (CellTiter 96 AQ_ueous_ One Solution Cell Proliferation Assay) was from Promega (Madison, WI).

### Ectopic expression and genetic silence of cPLA_2_α

Expression vector containing pCMV-cPLA_2_α or empty vector was stably transfected into DLD-1 cells using Lipofectamine^TM^ 2000 (Invitrogen, Melbourne, VIC, Australia). After 1 day of transfection, media was replenished with fresh medium containing selection antibiotic G418 at 1 mg/mL and cells were allowed to grow for 10 days. Isolated colonies were cultured in the presence of G418 (400 μg/mL). Two clones (Clone 15 and 18) were used for this study. Both show an increase in p-AKT. cPLA_2_α siRNA (TTG AAT TTA GTC CAT ACG AAA) and scramble control (GAA TTT CAA ACT CGA TAT AGT) were transfected into cells (10 nM siRNA duplexes) using HiPerfect Transfection Reagent (QIAGEN, Santa Clarita, CA) as described previously [16].

### Arachidonic acid release assay

Fatty acids were extracted from isolated cell pellets or culture media as described by Norris and Dennis [47]. A Xevo-Triple quadruple mass spectrometer (Waters, Micromass, UK) coupled to a Phenomenex Kinetex 1.7 μm C18 100A (2.1×150 mm) was used for arachidonic acid analysis. Standard curves were constructed using linear regression of the normalised peak areas of the analyte over internal standard (heptadecanoic acid) against the corresponding nominal concentrations of the arachidonic acid (See Supplemental method).

### 3-(4,5-dimethylthiazol-2-yl)-5-(3 carboxymethoxyphenyl)-2-(4-sulfophenyl)-2H-tetrazolium, inner salt (MTS) assay

CRC cells were plated in triplicate in 96 well plates. After 24 h, cells were incubated with Efipladib or DMSO (vehicle control) in 10% FCS-containing medium for 72 h prior to MTS assay. Stably transfected DLD-1 cells were grown in 10% FCS-containing RPMI 1640 for up to 4 days followed by MTS assay. The MTS assay was conducted as described previously [16]. Cell viability was independently monitored by Trypan Blue (Sigma-Aldrich) exclusion in parallel experiments.

### Cell cycle analysis

CRC cells were plated in triplicate in 6-well plates. After 24 h, cells adherent to plates were exposed to the indicated treatments. Cells were harvested, fixed in 70% v/v ice-cold ethanol, and incubated with propidium iodide (20 μg/mL) and RNase A (100 μg/mL) for 1 h in 37°C incubator. Cells containing propidium iodide-stained DNA were then assessed using FACSCalibur flow cytometer (BD Biosciences, Australia), and the percentage of cells in each phase of the cell cycle was analysed using Flowjo *v*8.0 (Tree Star, Ashland, OR).

### BrdU incorporation

CRC cells were incubated with BrdU at 10 μM in culture medium for 3 h before harvesting. Cells were then trypsinized, fixed in 10% v/v formalin, clotted in agarose gel, and processed for paraffin blocks. Sections of 5 μm thickness were cut and incubated at 60°C for 1 h, deparaffinized in xylene, re-hydrated in graded ethanol and distilled water, and subjected to antigen retrieval in Tris–EDTA solution using a microwave oven. Thereafter, the sections were treated with 2N HCl, blocked with 10% v/v house serum (Sigma-Aldrich) and incubated with anti-BrdU antibody overnight at 4°C. After being rinsed in Tris-buffered saline containing 0.05% Tween-20, the sections were sequentially labelled with a biotinylated secondary antibody and a Vectastain ABC kit from Vector Laboratories. Thereafter, the immunolabelling was visualized with 3,3′-diaminobenzidine tetrahydrochloride from Dako. Sections were scanned and analysed with an automated cellular imaging system (ACIS III, Dako, Denmark). The number of both BrdU-positive and negative cells over 10 randomly selected fields was determined and expressed as a percentage of positive cells in total number of cells.

### Xenografts assay

DLD-1 cells (2×10^6^) were implanted *s.c*. in the right flanks of 6 week male nude mice. Mice were randomly distributed into two groups once the tumour size reached 50 mm^3^ (7 mice/group). One treated with 200 μL of 20% v/v DMSO in PBS *i.p*. daily (as vehicle control); the other treated with Efipladib (10 mg/kg, *i.p.* daily) dissolved in DMSO and then diluted in PBS. Tumour growth was assessed every other day by caliper measurement of tumour diameter in the longest dimension (L) and at right angles to that axis (W). Tumour volume was estimated by the formula, L ×W ×W/2. Mice were sacrificed after 14 days of treatment and tumours were excised and the tissue distributed in two halves designated for Ki-67 immunostaining and immunoblotting. The protocol was approved by the Institutional Animal Care and Use Committee (Shanghai Jiao-Tong University).

### Immunoblotting

Cell lysates were prepared using RIPA buffer-1 (20 mmol/L Tris, 150 mmol/L NaCl, 1 mmol/L EDTA, 1 mmol/L EGTA, 1% v/v/Triton X-100, 2.5 mmol/L sodium PPi, 1 mmol/L h-glycerolphosphate), supplemented with protease inhibitor cocktail (cOmplete, Roche Diagnostics, Australia). Xenografts were excised from the hosts, homogenised in RIPA buffer-2 (50 mM Tris pH 7.4, 150 mM NaCl, 1% v/v Triton X-100, 1% w/v sodium deoxycholate, 0.1% w/v SDS) supplemented with the same protease inhibitor. Protein concentration was quantified using Bio-Rad Protein Assay (Bio-Rad, Hercules, CA). Cell lysates (50-100 μg protein) were separated on 8-10% SDS-PAGE and then transferred onto a nitrocellulose membrane; membranes were blocked with 5% w/v low fat skim milk in PBS containing 0.1% v/v Tween 20 for 1 h. Membranes were incubated overnight with primary antibodies at 4°C, followed by washing then probing with appropriate secondary antibodies coupled with peroxide and detected by enhanced chemiluminesence (Pierce, Rockford, IL). Gel-pro analysis *v*6.0 (Media Cybernetics, Bethesda, MD) was used for densitometric scanning and quantification.

### Immunohistochemistry

Tissue arrays were obtained from Outdo Biotech (Shanghai, China) with 120 individual cases of CRC and adjacent non-cancerous colon tissue from the same individual. Immunohistochemical staining was conducted using a DAKO EnVision+ System HRP as described previously [48]. An antibody raised in rabbit against cPLA_2_α (SC-438, 1:400 v/v) was left overnight at 20°C, an antibody in rabbit against phospho-cPLA­_2_α (SC-34391-p, 1:150 v/v) was applied at 37°C for 2 h. For Ki-67 immunostaining in xenograft recovered from mice, anti-Ki-67 was applied at 37°C for 2 h and purified rabbit-IgG (Dako, 1:60 v/v) was used as an isotype control.

### Imaging evaluation

cPLA_2_α immunostaining was assessed in a blinded manner using a light microscope (Olympus BX-50). The extent of staining was graded as 0 (<1%), 1 (1–20%), 2 (20–50%), 3 (50-75%) and 4 (>75%) in at least three independent fields using the same sample. The intensity of staining was assessed as: 0 (no staining), 1 (weak), 2 (moderate), and 3 (strong). The final score (range from 0 to 12) was obtained by multiplying the extent of staining with the intensity, and were defined as negative (0-3), + (4-6), ++ (7-9) and +++ (10-12). The images were acquired by software NIS-Elements F 3.0 (Nikon). The proportion of Ki-67 positive cells was quantified with ImageJ *v*4.2 (NIH).

### Statistical analysis

The statistical software SPSS version 14.0 was used for analysis. The scores of total and phospho-cPLA_2_α levels in CRC tissue were analysed by Wilcoxon signed rank test. The nonparametric Mann-Whitney U test was used to test whether the levels of cPLA_2_α and phospho-cPLA_2_α differ in gender, age, or M stage. Gamma regression was used to test the relationship between cPLA_2_α and T, N, TNM stage or differentiation. *In vitro* data were analysed by one-way ANOVA followed by multiple comparison tests. Xenograft growth was compared between groups by fitting a repeated measures covariate model, where the actual time measurements were viewed as a covariate. Two-tailed *P* value <0.05 was considered significant.

## SUPPLEMENTARY MATERIAL AND TABLE



## Abbreviation

AA: arachidonic acid
AKT: protein kinase B
cPLA2á: cytosolic phospholipase A2á
CRC: colorectal cancer
EGF: epidermal growth factor
EGFR: epidermal growth factor receptor
PI3K: phosphoinositide-3-kinase
PTEN: phosphatase and tensin homolog
VEGF: vascular endothelial growth factor
