# Supplementary material for: Targeting cytosolic phospholipase A2 α in colorectal cancer cells inhibits constitutively activated protein kinase B (AKT) and cell proliferation
Source: Oncotarget. 2014 Oct 28;5(23):12304–16. doi: 10.18632/oncotarget.2639 (PMC4322978; doi:10.18632/oncotarget.2639)
Supplement: Supplementary file 1 [file oncotarget-05-12304-s001.pdf]

Targeting cytosolic phospholipase $A_2\alpha$ in colorectal cancer cells inhibits constitutively activated Protein Kinase B (AKT) and cell proliferation

Supplementary Material

Arachidonic acid assay

Isolated cell pellets were mixed with 800 µL 1N HCl/MeOH (1:1 ratio) and 400 µL chloroform before vortexing for 1 min and then centrifuging at 1000 x g for 5 min to separate the two phases. The lower phase was then transferred to a fresh tube and the solvent was evaporated using a stream of nitrogen gas. Each sample was reconstituted in 250 µL MeOH containing 0.005% w/v butylated hydroxytoluene and 250 µL KOH (15% w/v) with vortexing. The solutions were then incubated at 37°C for 30 min. The samples were acidified (final pH < 3) with 1N HCl before adding 20 µL of 100 µg/mL heptadecanoid acid as an internal standard followed by extraction to hexane (see below). Media samples (2 x 250 µL from each sample) were diluted into a mixture of MeOH (400 µL), heptadecanoic acid (100 µg/mL, 10 µL) and HCl (1 M, 25 µL).

Both samples of pellet and media were then mixed with 2 mL hexane, vortexed for 30 s and centrifuged at 1000 x *g* for 5 min. The upper (hexane) layer was removed from the aliquots and combined into a 25 mL pear-shaped flask with a ground glass joint. The hexane separation was repeated and the combined hexane fractions were then evaporated under vacuum, reconstituted with 200 µL isopropanol and stored at -80°C before analysis using LC-MS. For quantification a standard calibration curve ranging from 0.01-20 ng/µL was constructed using purified arachidonic acid (Sigma-Aldrich, Cat. #: A3555) mixed with the same amount of heptadecanoid acid internal standard in all samples. The arachidonic acid concentration in each of the samples was then calculated based on the standard calibration curve.

A Xevo-Triple quadruple mass spectrometer (Waters, Micromass, UK) coupled to a Phenomenex Kinetex 1.7 µm C18 100A (2.1×150 mm) was used for analysis. Fatty acids (10 µL) were separated using a binary gradient program, at a flow rate of 0.1 mL/min (mobile phase A: 0.2% formic acid in water; and mobile phase B: isopropanol.) The gradient used for separation of fatty acid analytes was as follows: 0 min, 20:80 A/B; 3 min, 0:100 A/B; 6 min, 0:100 A/B; 7 min, 20:80 A/B. The mass spectrometer was operated in negative ESI mode with capillary voltage of 2.30 KV, cone voltage of 25 V and desolvation temperature of 500ºC. The collision induced dissociation (CID) gas used was argon. Before LC/MS analysis, the best fragments and collision energy for MS analysis of each fatty acid were obtained by acquiring mass spectra of the individual analytes in MS/MS mode under different collision energy ranging from 5 to 40 eV. Then the fragments with highest intensity were chosen at optimum collision energy (CE) for LC/MS analysis. Analytes were identified using the following mass transitions: for heptadecanoic acid, m/z 269.14 → 251.2 (CE 25 eV), for arachidonic acid *m/z* 303.3→ 59 (CE 20 eV), *m/z* 303.3→ 205 (CE 15 eV), *m/z* 303.3→ 259 (CE 15 eV), *m/z* 303.3→ 285 (CE 15 eV). Standard curves were constructed using linear regression of the normalised peak areas of the analyte (arachidonic acid) over internal standard (heptadecanoic acid) against the corresponding nominal concentrations of the analyte. Solutions of authentic standards (0.005 to 40 ng/µL of arachidonic acid containing 10 ng/µL of heptadecanoic acid) were prepared in a total volume of 1 mL isopropanol. Reference standards of arachidonic acid (purity ≥ 99.0%), heptadecanoic acid (purity ≥ 98%), LC-MS grade isopropanol and formic acid (purity ~ 98%), were all obtained from Sigma-Aldrich.

Supplemental Table1: Correlation of cPLA$_2$α expression/phosphorylation with clinical parameters (N=120 cases)

		cPLA$_2$α					p-cPLA$_2$α				
		-	+	++	+++	*P*	-	+	++	+++	*P*
Total cases	120	28	39	39	14		4	56	50	10	
Gender						*0.867*					*0.163*
Male	64	16	20	20	8		0	33	27	4	
Female	56	12	19	19	6		4	23	23	6	
Age						*0.597*					*0.285*
≤60	44	12	14	15	3		3	20	16	5	
>60	76	16	25	24	11		1	36	34	5	
TNM stage						*0.57*					*0.994*
Ⅰ	13	3	3	5	2		0	7	6	0	
Ⅱ	50	8	18	20	4		2	19	27	2	
III	46	16	14	13	3		2	25	14	5	
Ⅳ	11	1	4	1	5		0	5	3	3	
T stage*						*0.634*					*0.995*
Ⅰ	3	0	3	0	0		0	1	2	0	
Ⅱ	18	4	6	4	4		0	11	6	1	
III	85	21	28	29	7		3	38	37	7	
Ⅳ	8	3	1	4	0		1	5	2	0	
N stage						*0.075*					*0.358*
0	68	11	24	24	9		2	28	34	4	
1	39	12	12	10	5		1	23	9	6	
2	11	4	3	4	0		1	3	7	0	
3	2	1	0	1	0		0	2	0	0	
M stage						*0.099*					0.317
0	109	27	35	38	9		4	51	47	7	
1	11	1	4	1	5		0	5	3	3	
Degree of differentiation						*0.029*					*0.605*
Ⅰ	10	4	3	1	2		0	2	5	3	
Ⅱ	90	22	31	29	8		2	47	39	2	
III	20	2	5	9	4		2	7	6	5	

* Information on T stage was unavailable in six cases.
